# Linear magnetoresistivity in layered semimetallic CaAl_2_Si_2_

**DOI:** 10.1038/s41598-018-21102-9

**Published:** 2018-03-06

**Authors:** D. G. Costa, Rodrigo B. Capaz, R. Falconi, S. Strikos, M. ElMassalami

**Affiliations:** 1 Instituto de Física, Universidade Federal do Rio de Janeiro, Caixa Postal 68528, Rio de Janeiro, RJ 21941-972 Brazil; 2 Departamento de Química, Universidade Federal de Viçosa, Viçosa, Caixa Postal 216 Brazil; 3 División Académica de Ciencias Básicas, Universidad Juárez Autónoma de Tabasco, Cunduacán, Caixa Postal 86690 Mexico

## Abstract

According to an earlier Abrikosov model, a positive, nonsaturating, linear magnetoresistivity (LMR) is expected in clean, low-carrier-density metals when measured at very low temperatures and under very high magnetic fields. Recently, a vast class of materials were shown to exhibit extraordinary high LMR but at conditions that deviate sharply from the above-mentioned Abrikosov-type conditions. Such deviations are often considered within either classical Parish-Littlewood scenario of random-conductivity network or within a quantum scenario of small-effective mass or low carriers at tiny pockets neighboring the Fermi surface. This work reports on a manifestation of novel example of a robust, but moderate, LMR up to ∼100 K in the diamagnetic, layered, compensated, semimetallic CaAl_2_Si_2_. We carried out extensive and systematic characterization of baric and thermal evolution of LMR together with first-principles electronic structure calculations based on density functional theory. Our analyses revealed strong correlations among the main parameters of LMR and, in addition, a presence of various transition/crossover events based on which a *P* − *T* phase diagram was constructed. We discuss whether CaAl_2_Si_2_ can be classified as a quantum Abrikosov or classical Parish-Littlewood LMR system.

## Introduction

Recently, a vast array of materials were shown to exhibit extraordinarily high magnetoresistivity (MR) which is positive, nonsaturating and *linear-in-H* over wide ranges of magnetic field (10 Oe ≤ *H* ≤ 600 kOe) and temperature (4 ≤ *T* ≤ 400 K)^1–11^. These remarkable linear magnetoresistive (LMR)-bearing systems - with a huge potential for technological applications - are usually subdivided, based on the driving mechanism, into two broad classes. One class consists of classical Parish-Littlewood-type systems with spatial inhomogeneities arising from either macroscopic disorder or mobility (*μ*) fluctuations^11–13^. Here, simulations predict different behaviors for two limiting cases: Strong (${\rm{\Delta }}{\mu }/\langle {\mu }\rangle \gg 1$) and weak (${\rm{\Delta }}{\mu }/\langle {\mu }\rangle \ll 1$) disorder. For strong disorder, MR strength is proportional to mobility fluctuations (Δ*ρ*/*ρ* ∝ Δ*μ*), *H*_*X*_ ∝ (Δ*μ*)^−1^ (*H*_*X*_ is the crossover field from *quadratic into linear* behavior), longitudinal magnetoresistivity is weak and negative at high *H*, and LMR is large when electron and holes contribute equally (their effective 〈*μ*〉 = 0). The predictions for this high-disorder limit describe well the phenomenology of LMR in strongly inhomogeneous systems such as silver-doped chalcogenides^11–13^. On the other hand, in the weak disorder regime, LMR strength is proportional to the average mobility (Δ*ρ*/*ρ* ∝ 〈*μ*〉) and the crossover field is proportional to the inverse mobility (*H*_*X*_ ∝ 〈*μ*〉^−1^). Such predictions describe well the LMR in weakly inhomogeneous semiconductors with macroscopic spatial fluctuations in carrier mobilities^14^.

The other class consists of Abrikosov-type quantum systems^1,15–17^ such as the low carrier, small effective mass semimetals (with tiny carrier pockets near the Fermi surface) or the inhomogeneous almost zero-band-gap semiconductors with linear dispersion relation. The field and temperature ranges of Abrikosov quantum LMR effect are determined by the degree of confinement of charge carriers within the lowest Landau level. Within the assumption of a parabolic single band with effective mass m^*^, this leads to the two Abrikosov conditions for LMR. The first defines, in terms of the carrier density *n*, the crossover field *H*_*X*_:

1 $$H > {H}_{X}=\frac{\hslash c{n}^{\frac{2}{3}}}{e}\mathrm{.}$$

The second condition marks the upper temperature limit *T*_*A*_ for observing LMR:

2 $$T < {T}_{A}=\frac{e\hslash H}{{k}_{B}{m}^{\ast }c}\mathrm{.}$$

The above (classic or quantum) conditions are often used as criteria for identifying the underlying physical origin of the LMR effect. In this way, the LMR of the above-mentioned inhomogeneous semiconductors were considered to be classic, whereas the following materials were considered to be quantum LMR-bearing systems: elemental semimetal bismuth^4,5^, anisotropic layered metal LaSb_2_^6,7^, narrow-gap semiconductor InSb^3^, and layered semimetal graphite^18^.

In this work we present a novel example of a robust LMR in diamagnetic CaAl_2_Si_2_ which, although modest, manifests interesting features which cannot be straightforwardly classified as being driven by either a classic or a quantum mechanism. On the one hand, CaAl_2_Si_2_ is a layered compensated semimetal in which one electron pocket and three hole pockets coexist. As we shall see, our first-principle electronic structure calculations gave values of carrier densities (and its pressure dependence) that do not satisfy the above-mentioned Abrikosov first condition. On the other hand, various LMR features of CaAl_2_Si_2_ are irreconcilable with the classical Parish-Littlewood description as well: e.g., studied samples are single-phase polycrystals with no evidences supporting an appreciable inhomogeneity or distribution in its mobilities, and, furthermore, both LMR strength and crossover field *H*_*X*_ do not follow the classical predictions for a strong or weak disorder regimes.

In order to form a clear and consistent picture of LMR in CaAl_2_Si_2_ as well as to clarify the above-mentioned (quantum and classical) discrepancies, we systematically investigated thermal and baric evolution of LMR and perform extensive first-principles electronic structure calculations based on density functional theory (DFT). Our analyses reveal strong correlations among the main parameters of LMR and, in addition, a presence of various transition/crossover events based on which a *P* − *T* phase diagram is constructed. Finally, we discuss, based on our current understanding, whether LMR in CaAl_2_Si_2_ can be reconciled with currently available classical or quantum theories.

## Results and Analysis

**Figure 1:**
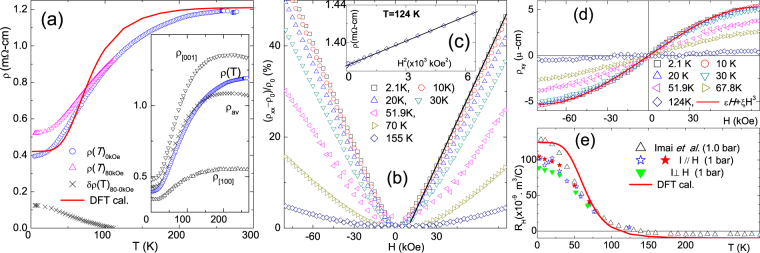
(**a**) Thermal evolution of *ρ*(*T*, 0 kOe) and *ρ*(*T*, 80 kOe) curves at ambient pressures measured on polycrystalline samples of CaAl_2_Si_2_ (this work). The solid red line (DFT cal.) represents the calculation based on Eqs 3, 5, 6. *δρ* (*T*)_80kOe_ = *ρ* (*T*, 80 kOe) − *ρ* (*T*, 0 kOe) is also shown. At ${T}_{A}\sim 100\,{\rm{K}}$, Δ*ρ* (*T*, 80 kOe, 1bar) drops to ∼3%. *Inset*: *ρ* (*T*, 0 kOe) is compared to *ρ*_[100]_ and *ρ*_[001]_ curves of single-crystal (ref.^21^). It is worth noting that residual resistivity in both monocrystalline or polycrystalline is within ∼m Ω-cm range. (**b**) Δ*ρ*_*xx*_(*H*)_*T*_ isotherms showing its even and *linear-in-H* character (indicated by solid black straight line) at lower *T* (the weak asymmetry character does not affect the conclusions reached in this work). (**c**) *ρ*_*xx*_(*H*)_124K_ exhibits the quadratic-in-*H* character at higher *T*. (**d**) The odd Hall resistivity *ρ*_*xy*_(*T*, *H*) is a sum of linear and cubic terms: for lower *H*, the linear approximation is in agreement with the measurements of ref.^19^. (**e**) Various *R*_*H*_(*T*) curves measured on polycrystalline samples (this work) and single-crystal (ref.^19^). The solid red line was calculated using Eq. 4.

Figure 1(a) shows representative resistivities, *ρ*(*T*, *H*), at ambient pressure and two values of magnetic field: Zero (blue circles) and 80 kOe (pink squares). Inset of Fig. 1(a) indicate that our polycrystalline *ρ*(*T*, 0 kOe) approximates the calculated powder-average of single-crystal measurements of Imai *et al*.^19^. Accordingly, it is inferred that our polycrystalline *ρ*(*T*, *H*) curves, as well as those of Imai *et al*.^19^, do reflect the intrinsic electronic properties of CaAl_2_Si_2_. As such, our conclusions will not be influenced by extrinsic scattering contributions from boundaries or defects.

Evidently, *ρ*(*T*, 0 kOe) increases with temperature until saturation (∼250 K) and, later on, a slight decrease at higher temperatures, revealing the semimetallic character of CaAl_2_Si_2_: Decreasing mobility and increasing carrier density compete, leading to a non-monotonic thermal evolution^19^. A temperature-dependent MR can already be observed in the difference curve of 0 and 80 kOe measurements (black crosses). Similar isofield measurements under various fields (not shown) reveal an unambiguous and robust MR. Likewise, the isotherm curves of Fig. 1(b) show that Δ*ρ*(*H*)/*ρ*_0_ is even, strong and *linear-in-H* for *T* < 100 K. In contrast, for *T* > 100 K, Δ*ρ*(*H*)/*ρ*_0_ is weak and exhibits the conventional *quadratic-in-H* behavior (see also Fig. 1(c)).

Figure 1(d) shows the expected *odd-in-H* Hall resistivity. The linear Hall coefficient, *R*_*H*_ shown in Fig. 1(e), demonstrates a strong dependence on temperature: it changes from positive to negative at *T* ≈ 120 K. Since CaAl_2_Si_2_ is a compensated semimetal (the electron density is equal to the hole density at all temperatures), this behavior is attributed to temperature dependence of carrier mobilities (see below)^19–21^.

**Figure 2:**
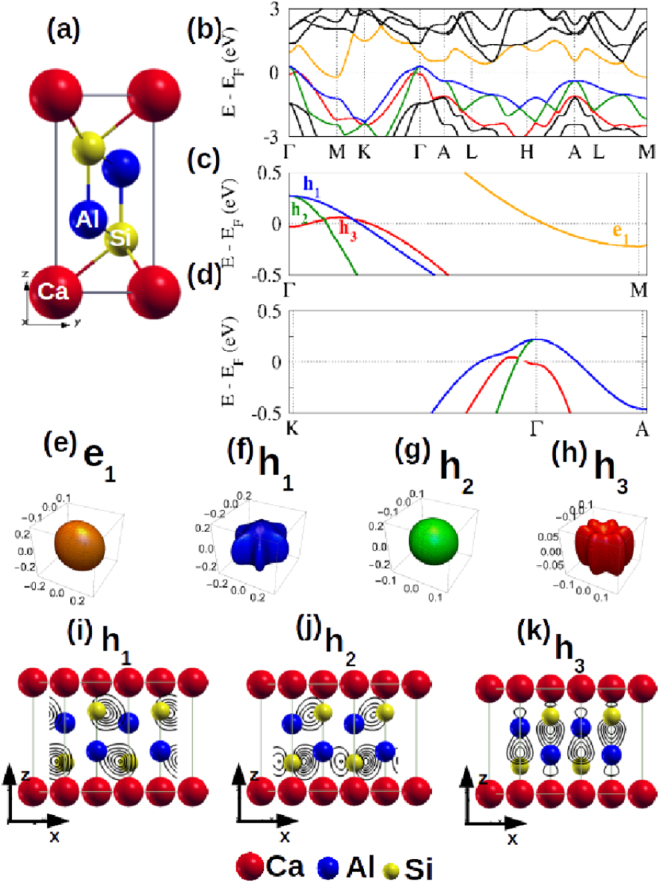
(**a**) Unit cell of CaAl_2_Si_2_. (**b**) Band structures within the neighborhood of *E*_*F*_. (**c**,**d**) Expanded view of the electron pocket along Γ − *M* direction (*e*_1_, orange) and the three holes, *h*_1_(blue), *h*_2_(green), *h*_3_(red) pockets along the Γ − *M*, Γ − *K* and Γ − *A* directions. The Fermi surfaces of the contributing pockets: (**e**) *e*_1_, (**f**) *h*_1_, (**g**) *h*_2_ and (**h**) *h*_3_. Note the difference in the scales of the various Cartesian systems. The projected |Ψ|^2^ of (**i**) *h*_1_, (**j**) *h*_2_ and (**k**) *h*_3_.

Before analyzing the LMR data in more detail, it is instructive to present our DFT-based electronic structure calculations. Fig. 2(a) shows the crystal structure of trigonal CaAl_2_Si_2_ (space group $P\bar{3}m1$) while Fig. 2(b) displays the band structure (Kohn-Sham eigenvalues) along selected symmetry directions, in good agreement with those of Refs^20,21^. Figure 2(c,d) show details of the band structure near the Fermi level. We highlight the existence of one electron pocket (*e*_1_) near the M point and three hole pockets (*h*_1_, *h*_2_ and *h*_3_) near the Γ point. Portions of the Fermi surface associated with these pockets are shown in Fig. 2(e–h). One sees that the *e*_1_ and *h*_2_ pockets are nearly spherical, *h*_1_ is quite anisotropic and *h*_3_ has a toroidal shape, as the top of the respective band is displaced from the Γ point. Noteworthy, the scales of the four k-space Cartesian systems are differently arranged such that *h*_3_ is conveniently visualized, otherwise this tiny hole pocket is nearly invisible. Finally, plots of |Ψ|^2^ for *h*_1_, *h*_2_ and *h*_3_ are shown in Fig. 2(i–k), revealing that hole pockets consist primarily of Al-Si bonding states^20,21^.

We proceed by comparing the experimental zero-field *ρ*(*T*) and *R*_*H*_(*T*) data of Fig. 1 with the corresponding curves based on our theoretical calculations. The resistivity and Hall coefficient are given, respectively, by:

3 $$\rho ={({\sigma }_{e}+\sum _{h}{\sigma }_{h})}^{-1}$$

4 $${R}_{{\rm{H}}}=\frac{\sum _{h}{\sigma }_{h}^{2}/{n}_{h}-{\sigma }_{e}^{2}/{n}_{e}}{ec(\sum _{h}{\sigma }_{h}^{2}+{\sigma }_{e}^{2})},$$

where *e* = *e*_1_ and *h* = *h*_1_, *h*_2_, *h*_3_. We obtain the carrier densities *n*_*e*_ and *n*_*h*_ and conductivities *σ*_*e*_ and *σ*_*h*_ directly from *k*-space integrations, starting from the conductivity tensor

5 $${\sigma }_{\alpha \beta }=2{e}^{2}{\int }_{BZ}\frac{{d}^{3}k}{2{\pi }^{3}}{\upsilon }_{\alpha }(\vec{k}){\upsilon }_{\beta }(\vec{k})\tau (\vec{k})(-\frac{df}{dE}),$$

where *f* is the Fermi-Dirac distribution, *υ*_*α*_ is the *α*-component of velocity and we perform an average over diagonal tensor components in order to comply with the polycrystalline character of our samples. For simplicity, we assume relaxation times to be *k*-independent but band-dependent: *τ*$(\vec{k})$ ≡ *τ*_*i*_ for *i* = *e*_1_, *h*_1_, *h*_2_, *h*_3_, with temperature dependence given by the Bloch-Gruneisen expression:

6 $${\tau }_{i}^{-1}(T)={\tau }_{i}^{-1}\mathrm{(0)}+{c}_{i}{(\frac{T}{{\theta }_{D}})}^{5}{\int }_{0}^{{\theta }_{D}/T}\frac{{z}^{5}}{({e}^{z}-\mathrm{1)(1}-{e}^{-z})}dz\mathrm{.}$$

The parameters *τ*_*i*_(0) and *c*_*i*_ are then fitted to the experimental *ρ*(*T*) and *R*_*H*_(*T*) data, using the reported^22^ *θ*_*D*_ ≈ 288 K.

The resulting fits of *ρ*(*T*) and *R*_*H*_(*T*), using the procedures outlined above, are shown as solid red lines in Figs. 1(a) and (e) respectively. Considering the few numbers of fitting parameters as well as the wide range of temperatures, it is assuring that the overall trends of both resistivity and Hall coefficient are satisfactorily revealed. In particular, the change in *R*_*H*_ from positive to negative (hole to electron conduction) with increasing temperature is quite well reproduced. We recall that charge compensation imposes that this change of behavior must arise from the temperature dependence of the mobilities of different bands^19–21^.

**Figure 3:**
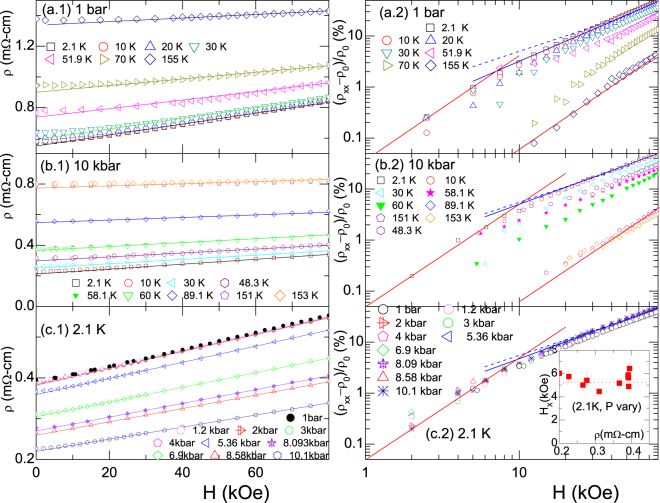
*H*-evolution of MR of CaAl_2_Si_2_. (**a.1,a.2**) various isotherms of *ρ*(*T*_0_, *H*, 1 bar); (**b.1,b.2**) various isotherms of *ρ*(*T*_0_, *H*, 10 kbar); and (**c.1,c.2**) various isobaric curves of *ρ*(2.1K, *H*, *P*_0_). All left-hand *ρ*(*T*, *P*) *vs H* curves were analyzed with linear fits [solid lines represent *m* = 1 of Eq. (7)]. In contrast, the right-hand plots are shown on a log-log scale. Here, fits to Eq. (7) reveal two limiting ranges: (i) For *H* < *H*_*X*_ or $T > {T}_{A}\approx {T}_{X}^{1{\rm{bar}}}$, fits are strictly *quadratic-in-T* [*m* = 2 in Eq. (7)], shown as solid red lines. (ii) For *H* > *H*_*X*_ and *T* < *T*_*A*_, we obtained $m\approx 1.1\sim 1.3$, shown as solid blue lines. The blue dashed lines represent the *m* = 1 limit which is evidently attained for higher values of *H* or *P*. *Inset*: A plot of *H*_*X*_(2.1 K, *P*) [obtained from the fits of panels (c.1–2)] *versus ρ*(2.1 K, *P*): Evidently, *H*_*X*_ is not proportional to *ρ* (see text).

Let us now analyze the thermal and baric evolution of LMR. Figure 3 shows the *H*-evolution of LMR for three sets of experimental conditions: (i) Different isotherms for *P* = 1 bar [Fig. 3(a.1,a.2)]; (ii) different isotherms for *P* = 10 kbar [Fig. 3(b.1,b.2)]; and (iii) different isobaric curves for *T* = 2.1 K [Fig. 3(c.1–c.2)]. At the right-hand side of Fig. 3(a.2, b.2, c.2), the same MR data (as in the left panels) are shown on log-log scales. This allow us to extract *H*_*X*_ in the usual manner. As an example, baric evolution of *H*_*X*_(*P*) at *T* = 2.1 K is shown in the inset of Fig. 3(c.2) (to be discussed in the next Section). Additionally, on a closer look, one occasionally observes a small deviations from linearity at *H* > *H*_*X*_ which can be expressed as

7 $$\frac{\Delta \rho (H,\,T,\,P)}{\rho \mathrm{(0,}\,T,\,P)}={\beta }_{m}(T,\,P){H}^{m},$$

where $m\approx 1.0\sim 1.3$ [see solid blue lines in Fig. 3(a.2,b.2,c.2)]. Deviations from linearity are possibly due to the multiband character of the Fermi surface (see below). Nevertheless, we find that as *H* (>40 kOe) is increased, *m* → 1 [see dashed blue lines in Fig. 3(a.2,b.2,c.2)]. Linearity is also manifested at higher pressures (*P* > 5 kbar).

**Figure 4:**
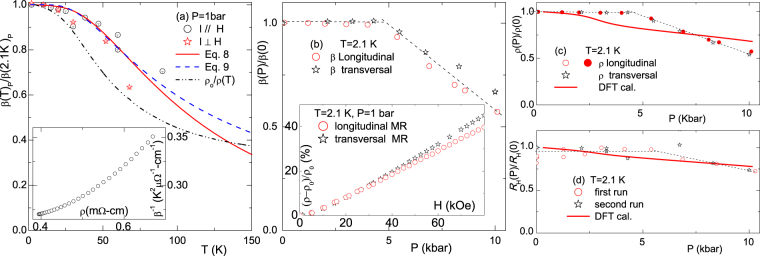
(**a**) Normalized $\frac{{\beta }({\rm T})}{{\beta }(2.1\,{\rm K})}$ curves measured at 1 bar. The red solid and blue dashed lines are fits using Eqs 8 and 9, respectively, wherein *c* = 80 K, *d* = 2 × 10^−5^ K^−2^, *t* = 140 K and *u*_*F*_ ≈ 0.2 *t* were obtained from the fits. The dashed-dot-dot black line represents $\frac{{{\rho }}_{{0}}}{{\rho }(T)}$ *versus T*. (**b**) Normalized $\frac{{\beta }(2.1\,{\rm K},\,P)}{{\beta }(0)}$ curve. *Inset*: Comparison of longitudinal and transversal MR at *T* = 2.1 K and *P* = 1 bar. (**c**) Normalized $\frac{{\rho }(2.1\,K,\,P)}{{\rho }(2.1\,K,\,{\rm{1}}\,{\rm{bar}})}$ curve. *Solid line*: calculated $\frac{{\rho }(0,\,P)}{{\rho }(0,\,0)}$ (see text). (**d**) Normalized $\frac{{R}_{H}(2.1\,{\rm{K}},\,P)}{{R}_{H}(2.1\,K,\,{\rm{1}}\,{\rm{bar}})}$ curves for two runs. *Solid line*: calculated effective $\frac{{R}_{H}(0,\,P)}{{R}_{H}(0,\,0)}$ (see text). In most cases, both longitudinal and transverse orientations are included. The dashed black lines in panels (b–d) are guides to the eye.

Figure 4(a,b) shows the thermal and baric evolution of the linear coefficient *β*_*m*_(*T*, *P*) extracted from Fig. 3(a.1,b.1,c.1) for *m* = 1. The ambient-pressure thermal evolution of normalized *β*_1_(*T*) [Fig. 4(a)] was analyzed in terms of three analytical expressions: (i) The empirical relation proposed by Takeya and ElMassalami^23^

8 $${\beta }_{{\rm{TM}}}(T)=b\frac{\tanh (c/T)}{(d\mathrm{.}{T}^{2}+\mathrm{1)}},$$

where *b*, *c* and *d* are fit parameters. (ii) Abrikosov’s expression^16^

9 $${\beta }_{{\rm{A}}}(T)=\frac{A\,\tanh \,(\frac{t}{T})}{\cosh \,(\frac{{u}_{F}}{T})/\cosh (\frac{t}{T})+1},$$

derived for a single-band layered semimetal where *u*_*F*_ is the chemical potential and *t* is the band half-width (both considered as fit parameters). (iii) The classical prediction in the weak disorder regime: $\beta \propto \langle \mu \rangle \propto {\rho }_{H\mathrm{=0}}^{-1}(T)$ with no fit parameters. Both Takeya-ElMassalami and Abrikosov’s expressions reproduce well the overall trend of *β*(*T*), even though CaAl_2_Si_2_ is a multiband system. (The denominator of Eq. 16 of this reference contains two identical terms. On comparison with the empirical Eq. 8^23^, one of the terms is considered to be cosh(tTtT). In that case, Eq. 8^23^ should tend to the high-TT limit 0.5A.tanh(t/T)0.25(u2F − t2)T−2 + 10.5A.tanh(t/T)0.25(uF2 − t2)T−2 + 1). In contrast, the classical prediction shows a strong deviation from the experimental data.

**Figure 5:**
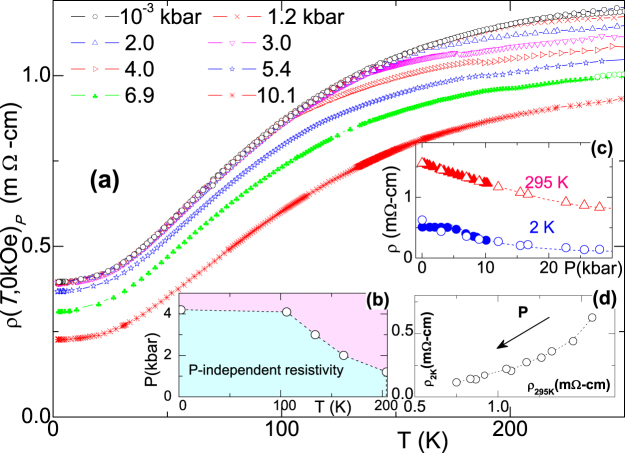
(**a**) Thermal evolution of zero-field, isobaric *ρ*(*T*, 0, *P* ≤ 10.1 kbar) curves showing the characteristic pair of ${P}_{X}^{T}$ and ${T}_{X}^{P}$. (**b**) *P* − *T* phase diagram as determined from the baric and thermal events observed in the main panel: Within the light-cyan region, all isothermal $\rho (T,H,P < {P}_{X}^{T})$ are *P*-independent. (**c**) The baric evolution of *ρ*(295 K, 0, *P*) and *ρ*(2.1 K, 0, *P*). (**d**) Correlation of *ρ*(2.1 K, 0, *P*) with *ρ*(295 K, 0, *P*) for different pressures. Noteworthy: in spite of the strong *P*-induced reduction of *ρ*(2.1 K, 0, *P*), no superconductivity was observed within the available *T*- and *P*-ranges.

The pressure dependence of *β* (2.1 K, *P*, *m* = 1), on the other hand, is shown in Fig. 4(b). One observes that *β* is nearly constant for *P* < *P*_*X*_ = 4.2 kbar and decreases linearly for *P* > *P*_*X*_: This suggests a critical or crossover event at *P*_*X*_. A similar crossover event is evident in *ρ*(*T*, *P*) curves of Fig. 4(c) and *R*_*H*_(2.1K, *P*) curves of Fig. 4(d). The temperature-dependence of *P*_*X*_ is also evident in the pressure-dependent *ρ*(*T*) curves of Fig. 5(a). The resulting *P*_*X*_(*T*) curve was used to construct the *P-T* phase diagram shown in Fig. 5(b). It is striking, and perhaps not accidental, that ${T}_{X}^{1{\rm{bar}}}$ almost coincides with *T*_*A*_ of Eq. 2 and with the temperature point at which *R*_H_(*T*) changes sign [Fig. 1(e)].

**Figure 6:**
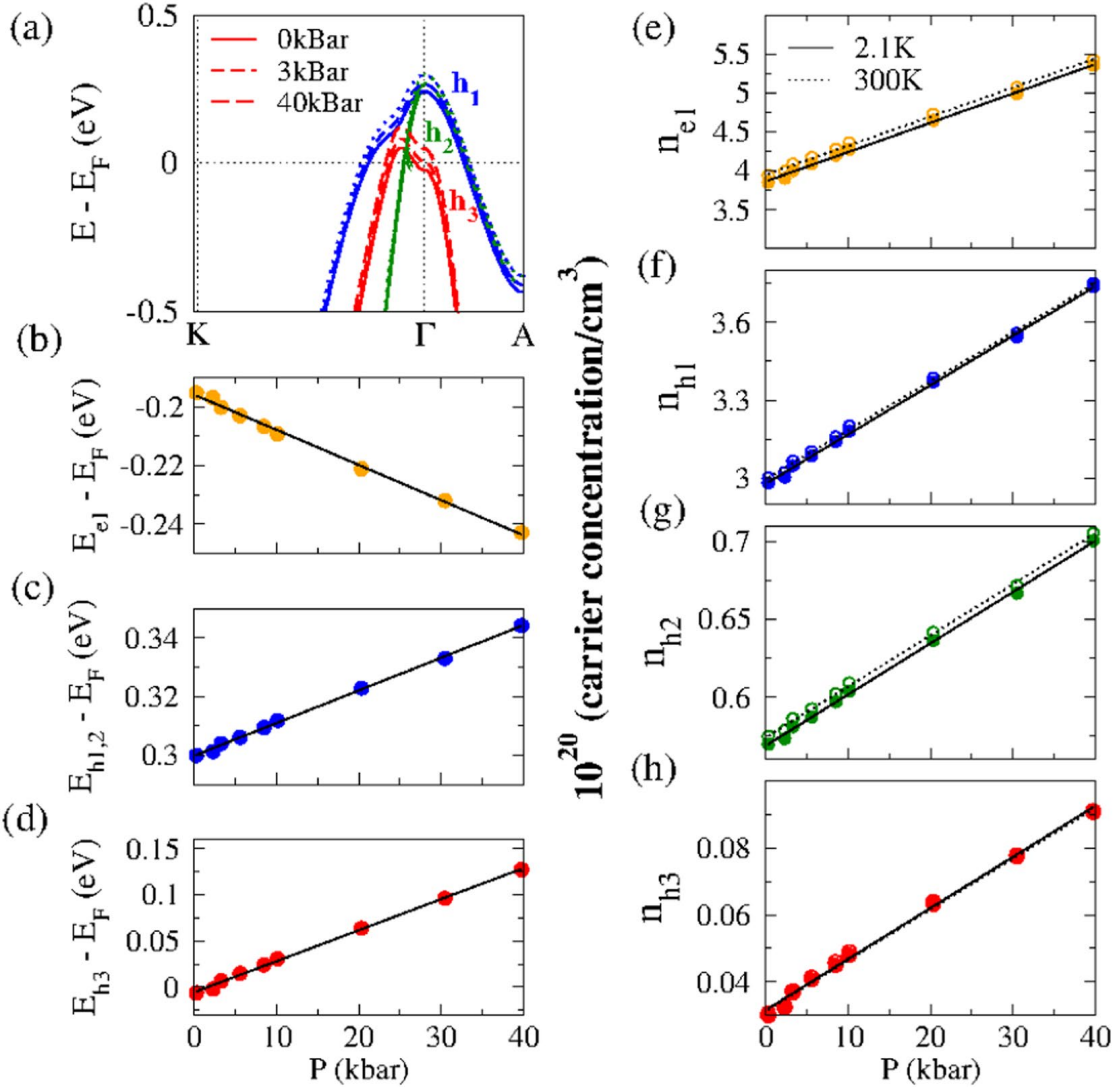
(**a**) Representative curves exhibiting the calculated baric influence on *h*_1_, *h*_2_ and *h*_3_. The baric evolution of (**b**) ${E}_{{e}_{1}}^{P}-{E}_{F}^{0{\rm{kbar}}}$ of *e*_1_, (**c**) ${E}_{{h}_{1},{h}_{2}}^{P}-{E}_{F}^{0{\rm{kbar}}}$ of the degenerate-at-Γ *h*_1_ and *h*_2_, and (**d**) ${E}_{{h}_{3}}^{P}-{E}_{F}^{0{\rm{kbar}}}$ of *h*_3_. The baric evolution of the carrier concentration of (**e**) *n*(*e*_1_), (**f**) *n*(*h*_1_), (**g**) *n*(*h*_2_) and (**h**) *n*(*h*_3_), each calculated for *T* = 2.1 and 300 K. Panels (*e*–*h*) demonstrate that all ${n}_{{h}_{1}}(P)$, ${n}_{{h}_{2}}(P)$, ${n}_{{e}_{1}}(P)$, and ${n}_{{h}_{3}}(P)$ are linear-in-*P* (within the limits of our conditions and resolution).

A manifestation of *P*_*X*_(*T*) crossover event is not reproduced by our DFT calculations. As evident from Fig. 4(c,d), our model calculations give, roughly, a linear decrease of both *ρ*(2.1 K, *P*) and *R*_*H*_(2.1 K, *P*); here, *τ*_*i*_(0) are considered to be pressure-independent. Further insight can be obtained by calculating the pressure-dependent changes in band structure and carrier density. As shown in Fig. 6(a), the top of each hole pocket at Γ rises as *P* increases, while the bottom of the electron pocket near *M* sinks (not shown). The pressure shifts of the various bands are roughly linear with pressure in this low-*P* regime, as shown in Fig. 6(b,c and d). As a result of these band-shifts, the calculated DOS at the Fermi level increases monotonically with *P*. Also, Fig. 6(e–h) indicate a linear baric evolution of the calculated carrier concentration for each individual band, which is consistent with the overall decrease of both *ρ*(*P*) and *R*_*H*_(*P*), but disagrees with the surge of *P*_*X*_ event and the almost constant *H*_*X*_(P) *vs ρ*(*P*) shown in Inset of Fig. 3(c.2). For explanation of the above-mentioned discrepancy, we speculate that an increase in pressure not only moves, as an example, the *h*_3_ pocket upwards in energy but also changes its topology from toroidal to spheroidal shape at roughly 5 kbar. The implications of such unusual Fermi surface topologies on LMR is a topic of future interest.

Concerning the *P*_*X*_(*T*) boundary line, our ambient-pressure, temperature-dependent crystal structure analysis (see supplementary materials, SM) rules out any structural phase transition at low temperatures. Moreover, based on our DFT calculations, we can also rule out any sort of crystal structural phase transition or any other unusual structural behavior at such small pressures: Indeed, our calculations show that lattice constants exhibit linear pressure-induced reduction (see Fig. S1 in SM), though anisotropic due to the layered character of the $P\bar{3}m1$ structure. Nevertheless, our calculations predict an occurrence of structural phase transitions at much higher pressures, well above our present pressure ranges^24^.

**Figure 7:**
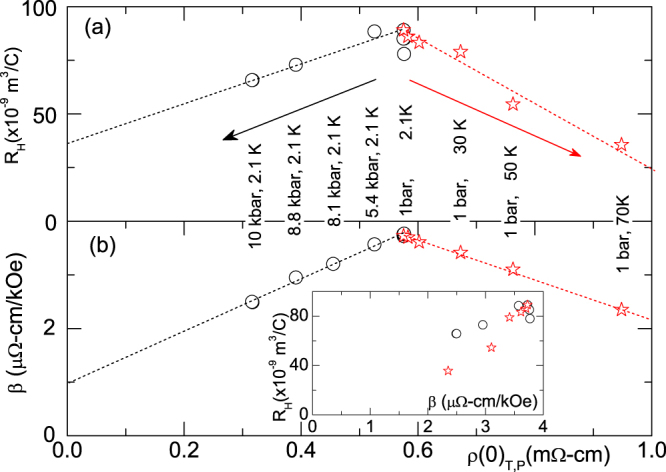
Correlation of *R*_*H*_ and *β* with *ρ*(*T*, 0, *P*): *circles* indicate the isothermal baric evolution while *stars* denote the isobaric thermal behavior. (**a**) *R*_H_ as a function of *ρ*(0). (**b**) *β* as a function of *ρ*(0). Dashed lines are guides to the eye. On increasing *P* or *T* (represented by the left- and right-ward arrows, respectively), both *β* and *R*_H_ are reduced; *ρ*(0), on the other hand, is reduced by *P* while increased by *T*. *Inset*: Correlated trend of *R*_*H*_ and *β*: a decreasing trend when any of *P* or *T* is increased.

The possibility of probing separately the pressure and temperature dependences of *β* as well as *ρ*_0_ provides two independent handles for verifying, in CaAl_2_Si_2_, the Kohler’s rule^2,25^ which states that Δ*ρ*_*xx*_/*ρ*_0_ is a function of *H*/*ρ*_0_ and this, for LMR, yields Δ*ρ*/(*Hρ*_*o*_) = *β*_1_ ∝ 1/*ρ*_*H*=0_ (here, in this work, *ρ*_0_ = *ρ*_*H*=*o*_ is a temperature-dependent zero-field resistivity, not a residual, *T* = 0, quantity). As known, this rule is a powerful test of whether a single-band semiclassical description (using a single relaxation time *τ*) can explain the evolution of the magnetotransport properties. Inset of Figure 4(a) shows that Kohler’s rule is strongly violated in CaAl_2_Si_2_. As a matter of fact, pressure- and temperature-induced variations of *ρ*_*H*=0_ (Fig. 7) produce *opposite* trend in *β*, meaning that LMR in this material shows a more complex dependence on carrier density (tuned by pressure) and relaxation time (tuned by temperature) than predicted by Kohler’s rule. It is worth adding that similar Kohler-like correlations among the parameters of LMR can be seen in other LMR-bearing systems such as *AM*_2_B_2_ and *A*_3_Rh_8_B_6_ (*A* = Ca, Sr; *M* = Rh, Ir) series^23^, Bi thin films^5^, InSb^3^, Ag_2+*δ*_*X* (*X* = Se, Te)^2,8,9^, LaSb_2_^6,7^, graphene^26^, graphite^27^, GaAs-MnAs^28^ and BaFe_2_As_2_^29^.

Finally, although CaAl_2_Si_2_ is a compensated semimetal similar to Bi^4,5^ or WTe_2_^30,31^, its modest LMR is distinctly different from their extremely high MR: It is recalled that Bi (WTe_2_) manifests a typical *linear-in-H* (*quadratic-in-H*) MR. As far as comparison with quantum LMR-bearing semimetals is concerned, Abrikosov relation

10 $${\rm{\Delta }}\rho =\frac{{N}_{i}}{\pi {n}_{eff}^{2}ec}H$$

(all terms have their usual meanings), it is recalled that the impurities *N*_*i*_ and the effective charge carrier *n*_*eff*_ are material-dependent quantities: This suggests that care should be exercised when comparing the magnetoresistivities of different materials.

## Discussion and Summary

Based on the above-mentioned thermal, field and baric evolution of LMR of CaAl_2_Si_2_, we now discuss whether this effect can be explained with the classical Parish-Littlewood or quantum Abrikosov models.

Let us first discuss, within the classical model, whether the linearity as well as the thermal and baric evolution of MR originate from sample inhomogeneity or spatial fluctuations of conductivities^11–13^. As mentioned above, classical-model simulations provide predictions in two limits of disorder: The strong $\frac{{\rm{\Delta }}{\mu }}{{\mu }} > 1$ and weak $\frac{{\rm{\Delta }}{\mu }}{{\mu }} < 1$ cases. It is recalled that our *ρ*(*T*) curve shown in Fig. 1 reproduces, in magnitude and thermal evolution, the weighted average of *c-axis ρ*_[001]_ and *a-axis ρ*_[100]_ curves^19^ and, furthermore, there is only a weak anisotropy of 1.5 < $\frac{{{\rho }}_{[001]}}{{{\rho }}_{[100]}}$ < 2.5. These features exclude the possibility of strong disorder or in-homogeneity. There are two additional arguments that support such an exclusion: (i) Based on simulations^11–13^, one expects a negative and weak longitudinal MR. In contrast to such an expectation, the inset of Fig. 4(b) shows a positive and equally strong longitudinal LMR (as compared to the transversal one). (ii) One also expects a maximum in the magnitude of transverse LMR at temperature range (∼120 K) where *R*_*H*_ → 0 (both positive and negative charge carriers are contributing)^11–13^. In sharp disagreement, LMR within this region vanishes, being substituted by a *quadratic-in-H* behavior.

For the weakly disordered case $(\frac{{\rm{\Delta }}{\mu }}{{\mu }} < 1)$^14^, let us recall the above-mentioned clear violation of Kohler’s rule; specifically Inset of Fig. 4(a) indicates that $\frac{1}{{{\rho }}_{H=0}}$ does not follow *β*; rather, as the temperature is increased, *ρ* is also increased due to a decrease in relaxation time, however the predicted *β* is much decreased, more than the measured one. On the other hand, Figs 4(b–d) and 7(b) indicate that on increasing pressure (*ρ*_*H* = 0_ decreases due to an increased carrier density), the measured *β* is *also* decreased. The failure of another weak-limit prediction (namely, *H*_*X*_ ∝ *ρ*_*H*=0_)^14^ is shown in the inset of Fig. 3(c.2). Based on these two failure, we conclude that CaAl_2_Si_2_ can not be described within the weakly disordered model (just as the failure of the strongly disordered case). Therefore, the only possibility that LMR in CaAl_2_Si_2_ has a classical origin would be that it falls into some intermediate-disordered regime hitherto not addressed by numerical simulations; mind that even such a limit is ruled out by the satisfactorily agreement between polycrystalline and averaged monocrystalline resistivities shown in Fig. 1(a).

Let us now discuss a quantum scenario for LMR, noting that CaAl_2_Si_2_, being a semimetal with small pockets near the Fermi surface, is a suitable candidate and that this candidacy can be verified by testing Abrikosov’s conditions, Eqs 1–2, after substitution of the involved parameters that can be obtained from experiments and DFT calculations. For checking the first condition (Eq. 1), we considered, based on Fig. 1(b), *H*_*X*_ ≈ 10 kOe; then Eq. 1 gives *n* ≈ 6 × 10^16^ cm^−3^. This value is four orders of magnitude lower than the calculated carrier densities of the hole and electron pockets which, at zero temperature and pressure, are ${n}_{{e}_{1}}=3.68\times {10}^{20}$ cm^−3^,  ${n}_{{h}_{1}}=3.08\times {10}^{20}$ cm^−3^, ${n}_{{h}_{2}}=0.57\times {10}^{20}$ cm^−3^ and ${n}_{{h}_{3}}=0.03\times {10}^{20}$ cm^−3^. Thus, DFT calculations suggest that the first condition is not satisfied, although one must realize that DFT band energies represent an approximation of the true quasiparticle energies. Then it would be possible that our band energies are shifted by a few tenths of eV with respect to the true values. This could have dramatic consequences on the values of carrier density, possibly bringing them (particularly *h*_3_) into closer agreement with Abrikosov’s first condition. It is possible to carry out a comparison of the calculated rate of pressure-induced increase of charge concentration with the rate of pressure-induced decrease of LMR effect: such consideration should await an improved DFT analysis of the *P*_*X*_(*T*) events.

The second Abrikosov condition describes the survival of LMR up to *T*_*A*_, which is *H*-dependent. From Fig. 1(b), we estimate *T*_*A*_ ≈ 52 K for *H* ≈ 40 kOe, which gives m^*^ ≈ 0.1*m*_*e*_. Based on the carrier density dependence on Fermi energy (shown in Fig. 6), we estimate average effective masses for the various bands as ${m}_{{e}_{1}}^{\ast }=0.9{m}_{e}$, ${m}_{{h}_{1}}^{\ast }=0.5{m}_{e}$, ${m}_{{h}_{2}}^{\ast }=0.2{m}_{e}$ and ${m}_{{h}_{3}}^{\ast }=0.08{m}_{e}$. The effective mass for *h*_3_ holes approaches the predicted value from Abrikosov’s condition, the others being slightly heavier but within the correct order of magnitude. We emphasize, however, that the electronic structure of CaAl_2_Si_2_ shows multiple and nonspherical carrier pockets; as such it departs considerably from the simplified single, parabolic band situation considered by Abrikosov. Evidently, a complete description of the novel LMR in CaAl_2_Si_2_ within the current quantum model calls for further theoretical developments or extension so as to treat the case of multiple bands with non-spherical Fermi surfaces. It is also evident that a better verification of the Abrikosov conditions would be effected when we extend our DFT analysis so as to yield an improved determination of carrier densities and effective masses.

In summary, this work reports on a new LMR-bearing material, namely the layered, compensated, semimetallic CaAl_2_Si_2_. Extensive characterization of the baric and thermal evolution of this LMR was carried out and their analysis revealed strong correlations among the main parameters of LMR and, in addition, a presence of various transition/crossover events based on which a *P* − *T* phase diagram was constructed. First-principles DFT calculations provided a basic structural and electronic characterization of this compound, including pressure influence on the band structure. Based on these calculations and together with the thermal and baric evolution of LMR, we argue that LMR of CaAl_2_Si_2_ is novel and does not fit the classical or quantum descriptions in their standard form, thus calling for further theoretical developments. Further analysis is underway to provide a better characterization of LMR, a further refinement of the theoretical calculations of thermal and baric evolution of charge densities and a further analysis of the mechanism behind the *P* − *T* phase diagram, in particular, the anomalous critical/crossover behavior observed in the baric evolution of LMR and Hall parameters.

## Methods

Polycrystalline samples of CaAl_2_Si_2_ were synthesized via an argon arc-melt method^19,22,32^. For compensation of possible loss of Ca, the starting weight of Ca was augmented by an excess of approximately 10 percent. Samples, once synthesized, are stable in air over at least one year. X-ray and neutron powder diffraction analyses (see SM) confirmed the stoichiometry as well as the single-phase structure with lattice parameters which are in excellent agreement with earlier reports^19,22,32,33^. Two pressure cells were used for measuring a four-point DC magnetoresistance within 2 ≤ *T* ≤ 300 K and *H* ≤ 90 K. One cell (up to 10 kbar) uses extraction naphtha as a pressure-transmitting fluid and a heavily doped bulk *n*-InSb single crystal as a pressure gauge. On this cell, both transverse and longitudinal resistances were studied and, in addition, Hall voltage was measured under the same experimental conditions. The second pressure cell (<30 kbar) uses Fluorinert and an extrapolated curve for pressure calibration; with this cell, only transverse resistivity was studied. In all cases, phonon contribution to *ρ*_T,P_ is taken to be *H*-independent.

Our theoretical calculations were performed within the first-principles Density Functional Theory under the generalized gradient approximation of Perdew, Burke, and Ernzerhof^34^. The Quantum Espresso *ab initio* simulation package was used to perform all calculations^35^. Kohn-Sham orbitals were expanded in a plane-wave basis set with a kinetic energy cutoff of 50 Ry (300 Ry for the density). First Brillouin Zone integrations were performed with 10 × 10 × 10 *k*-point sampling^36^. Pressure-dependent calculations were performed with target pressure covering the range 0 ≤ *P* ≤ 40 kbar; for each pressure, a full optimization of the unit cell and atomic positions were performed until all forces are below 10^−4^ Ry/Bohr and with an energy tolerance of 10^−6^ Ry. Finally for the analysis of the pressure-induced changes in the band structure, the *k*-space integration was performed by the improved tetrahedron method with a 24 × 24 × 24 mesh^37^.

## Electronic supplementary material

Supplementary information
