# Supplementary material for: Linear magnetoresistivity in layered semimetallic CaAl2Si2
Source: Sci Rep. 2018 Mar 6;8:4102. doi: 10.1038/s41598-018-21102-9 (PMC5840406; doi:10.1038/s41598-018-21102-9)
Supplement: Supplementary file 1 — Supplementary information [file 41598_2018_21102_MOESM1_ESM.pdf]

Supplementary Material: Linear magnetoresistivity in layered semimetallic $CaAl_2Si_2$

D. G. Costa[1,2], Rodrigo B. Capaz[1], R. Falconi[3], S. Strikos[1], and M. ElMassalami[1,*]

[1]Instituto de Física, Universidade Federal do Rio de Janeiro, Rio de Janeiro, Caixa Postal 68528, Brazil
[2]Departamento de Química, Universidade Federal de Viçosa, Viçosa, Caixa Postal 216, Brazil
[3]División Académica de Ciencias Básicas, Universidad Juárez Autónoma de Tabasco, Cunduacán, Caixa Postal 86690, México
[*]massalam@if.ufrj.br

ABSTRACT

A Baric and thermal evolution of structural parameters $CaAl_2Si_2$

Theoretical calculations of baric evolution of the optimized unit-cell dimensions as well as ambient-pressure X-ray and neutron diffraction analysis indicated that the hexagonal unit cell and the space group ($P\bar{3}m1$) are maintained throughout the studied pressure and temperature ranges.

The theoretical analysis, on the one hand, indicated a systematic pressure-induced linear reduction of the lattice parameters (see Fig.S1). Evidently, the reduction of the c-parameter is much stronger than that of the a-parameter ($\frac{\Delta c}{c_o} \approx \frac{2\Delta a}{a_o}$). This anisotropic feature is also manifested in the single-crystal resistivity [Ref.(1)] and in the electronic arrangement of h_1, h_2, and h_3 pockets of Figs. 2 and 6 of the main text; it is consistent with the fact that this three-dimensional structural network is formed by linking the Al_2Si_2 layers through the Si-Si bonds: Such inter-layer X-X bonding is common among the layered AM_2X_2 structures (A=alkaline earth, M=transition metal, X from group 14 and 15 elements; see Refs. (2; 3)).

The diffraction studies, on the other hand, exhibited diffractograms similar to those reported in Ref.(1; 4; 5; 6). The evolution of their structural parameters are shown in Table S1. Our structural analysis confirm that, except for a weak thermal contraction, there is no anomalous change in either atomic positions or lattice parameters: Absence of any thermal induced structural phase transition or crossover within the studied range of temperature and, from above paragraph, pressure.

References

1. Imai, M., Abe, H. & Yamada, K. Electrical properties of single-crystalline $CaAl_2Si_2$. *Inorg. Chem.* **43**, 5186–5188 (2004).

2. Hoffmann, R. & Zheng, C. Making and breaking bonds in the solid state: the thorium chromium silicide $ThCr_2Si_2$ structure. *J. Phys. Chem.* **89**, 4175–4181 (1985).

3. Berry, N. *et al.*. Superconductivity without fe or ni in the phosphides $BaIr_2P_2$ and $BaRh_2P_2$. *Phys. Rev. B* **79**.

4. Kranenberg, C., Johrendt, D. & Mewis, A. Investigations about the stability range of the $CaAl_2Si_2$ type structure inthe case of ternary silicides. *Z. Anorg. Allg. Chem.* **11**, 1787–1793 (1999).

5. Kuo, Y. K. *et al.*. The effect of Al/Si ratio on the transport properties of the layered intermetallic compound $CaAl_2Si_2$. *J. Phys.: Cond. Mat.* **19**, 176206–176210 (2007).

6. ElMassalami, M., Paixão, L. S. O. & Chaves, F. A. B. Resistivity studies on the layered semi-metallic $CaAl_2Si_2$: evaluating its temperature-, field- and pressure-dependence. *J. Phys.: Cond. Mat.* **23**, 245701–245705 (2011).

7. Wang, L. *et al.*. Inorganic crystal structure data base. http://www.fiz-karlsruhe.de/de/leistungen/kristallographie/icsd.html (2009).

8. Gladyshevsky, E. I., Kripyakevich, P. I. & Bodak, O. I. Crystal structure of $CaAl_2Si_2$. *Ukr. Fiz. Zh.* **12**.

9. Huang, G. Q., Liu, M., Chen, L. F. & Xing, D. Y. Electronic structure and electron-phonon interaction in $CaAl_2Si_2$. *J. Phys.: Cond. Mat.* **17**, 7151–7157 (2005).

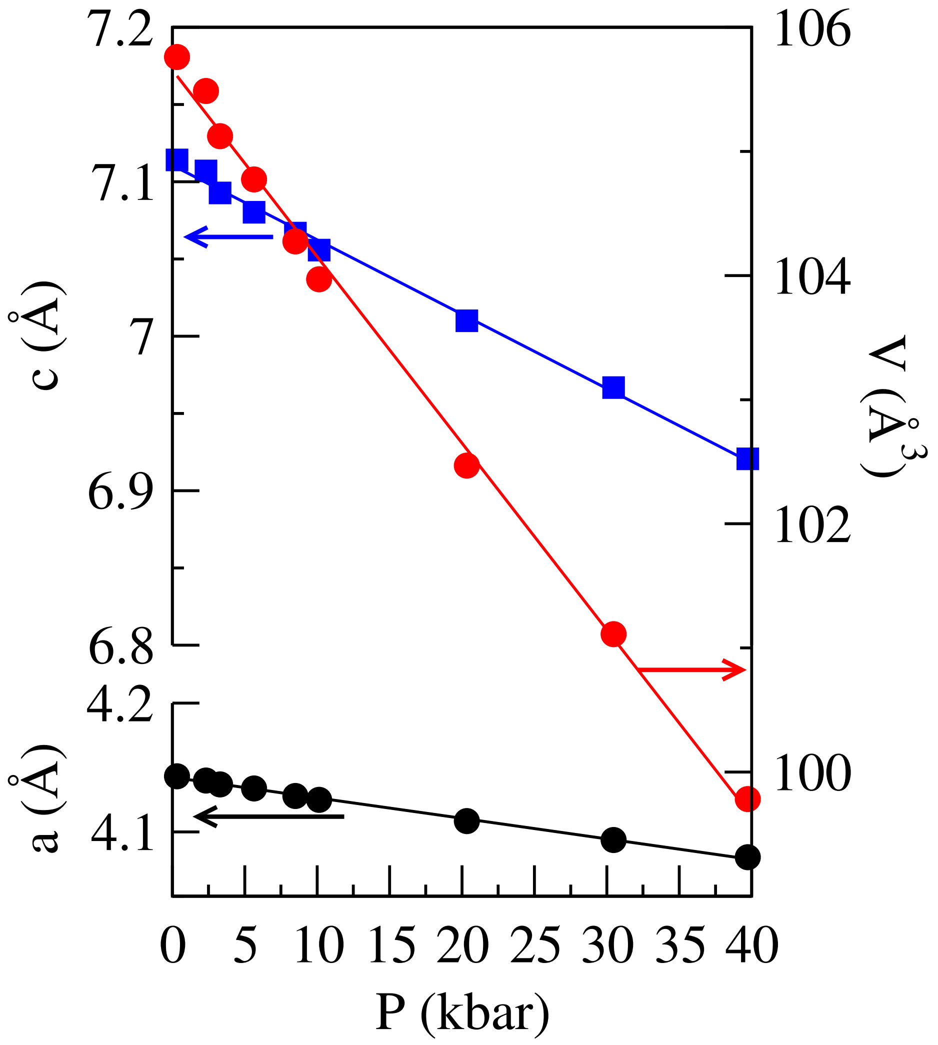

Figure S1. Calculated baric evolution of the optimized unit cell dimensions: $a = 4.136 - 0.0014P$ Å; $c = 7.102 - 0.0041P$ Å, $V = 105.27 - 0.126P$ Å^3 (P in kbar). As evident, there is a linear-in-P evolution, with no trace of any event at $P_X \approx 4$ kbar: accordingly, the P_X event (see main text) can not be attributed to an induced structural phase transition. Similarly, no ambient-pressure structural instability can be inferred from the analysis of low-temperature diffractograms.

Table S1. The lattice parameters (a, c, V, z_{Al} and z_{Si}) as measured experimentally and theoretically calculated from optimized DFT calculations. XRD (ND) denotes the lattice parameters as obtained from X-ray diffraction at 300K and 85 K (neutron diffraction at 300 and 1.5 K). For all, experiments and theory, the following structural properties are the same as the ones given in Ref. 7: space group is $P-3m1$ with a hexagonal unit cell having α = 90°, γ = 120° and Ca at (0,0,0), Al at (1/3,2/3,0.63(1)) and Si at (2/3,1/3,0.27(1)). Similarly, the thermal coefficients and occupation numbers are fixed to those of Ref. 7. In contrast lattice parameters are temperature and pressure dependent.

Compound	XRD (300K)	XRD(300K)	XRD(85K)	ND(300K)	ND(1.5K)	cal.	cal.
Ref	[1,7,8]	this work	this work	this work	this work	[9]	this work
a (Å)	4.141	4.140(1)	4.137(1)	4.140(1)	4.134(1)	4.142	4.14
c (Å)	7.133	7.127(1)	7.115(1)	7.130(1)	7.106(1)	7.137	7.11
V (Å^3)	105.929	105.798(15)	105.472(10)	105.814(11)	105.166(8)	106.039	105.536
